# Vision Related Quality of Life in Patients with Keratoconus

**DOI:** 10.1155/2014/694542

**Published:** 2014-04-29

**Authors:** Sevda Aydin Kurna, Ahmet Altun, Tugba Gencaga, Sezen Akkaya, Tomris Sengor

**Affiliations:** ^1^Fatih Sultan Mehmet Education and Training Hospital, Ophthalmology Clinics, Omerli Park 2 Sitesi No. 2, Kadirova Mevkii, Omerli, Cekmekoy, 34797 Istanbul, Turkey; ^2^Bilim University-Florence Nightingale Hospital, Ophthalmology Clinics, 34797 Istanbul, Turkey

## Abstract

*Purpose.* The purpose of this study is to evaluate the vision related quality of life in patients with keratoconus by using the National Eye Institute Visual Function Questionnaire-25 (NEI-VFQ-25). *Methods.* Thirty patients presented with keratoconus (keratoconus group) and 30 healthy patients (control group) were included in this study. Twenty patients were using rigid gas permeable and 10 patients were not using contact lenses in keratoconus group. High and low contrast visual acuity and mean *K* values of the patients were recorded. Each subject completed the 25-item NEI-VFQ-25. *Results.* All subscales of NEI-VFQ-25 were lower in the keratoconus patients. The difference was more evident in the subscales of general vision, ocular pain, near vision, vision-specific mental health, vision-specific role difficulties, and peripheral vision (*P* < 0.05). Overall composite score was 75.2 ± 17.2 in the keratoconus group and 93.2 ± 5.6 in the control group (*P* = 0.00). Contact lens wearers had higher best corrected visual acuity in comparison with noncontact lens wearers (*P* = 0.028). Patients with low visual acuity (logMAR > 0.4) in the better eye had lower distance vision, social functioning, mental health, and role difficulties. Meanwhile, patients with low visual acuity (logMAR > 0.4) in the worse eye had lower general health scores (*P* < 0.05). *Conclusions.* Vision related quality of life was worse in keratoconus patients. Success in the contact lens usage and maintaining higher visual acuity may improve vision related quality of life.

## 1. Introduction

Keratoconus is a progressive, bilateral asymmetric, noninflammatory corneal ectasia with an incidence of 1 per 2,000 in the general population [1]. While keratoconus mainly affects young adults, other eye diseases that affect vision such as glaucoma and macular degeneration have a much later onset. The corneal thinning induces irregular astigmatism, myopia, and protrusion, leading to mild to marked impairment in the quality of vision. Keratoconus patients report ocular discomfort and poor vision typically treated with contact lenses or spectacles [1, 2].

Health related quality of life (HR-QoL) measures functioning and well-being in physical, mental, and social health realms of life and reflects the influence of a broad range of health conditions simultaneously. The National Eye Institute (NEI) sponsored the development of the National Eye Institute-Vision Function Questionnaire (NEI-VFQ) with the goal of creating a survey that would measure the dimensions of self-reported vision targeted health status that are most important for persons who have chronic eye diseases. VFQ-25 is the product of an item reduction analysis of the longer field test version of the survey called the 51-item NEI-VFQ [3].

NEI-VFQ-25 assesses vision related quality of life multidimensionally by several subscales such as general, near, distance and color vision, role limitations, dependency, mental health, and social function. They have been validated by a variety of studies showing they are useful tools in assessing vision-specific quality of life [4–8]. NEI VFQ-25 is demonstrated to be sensitive to the influence of age related macular degeneration (AMD) [9, 10], glaucomatous field loss [11], cataract [12], Behcet uveitis [13], after penetrating keratoplasty for keratoconus [14], after retinal detachment surgery [15], vitrectomy [16], diabetic retinopathy [17], strabismus [18], multiple sclerosis [19], osteoporotic fractures, and low vision from any cause [20]. The correlations with clinical markers of disease severity provide evidence of clinical validity for the measure. Significant impairment in vision related quality of life (VR-QoL) with average scores comparable to age related macular degeneration has been shown by the collaborative longitudinal evaluation of keratoconus study group [21].

NEI-VFQ-25 evaluates not only visual function and limitations in daily activities related to impaired visual function but also the impact of ocular disease on patients' lives from various standpoints. Apart from clinical evaluation, anatomical success, and visual acuity, other aspects of visual outcome should also be considered in the keratoconus patients. For this reason, to understand the effect of the keratoconus disease on VR-QoL and to evaluate disease from the perspective of these younger-age patients in their active years should be an important concern. The purpose of this study was to evaluate the VR-QoL and visual function in the keratoconus patients by using the NEI-VFQ-25.

## 2. Methods

Consecutive patients with keratoconus attending to the cornea and contact lens department of Fatih Sultan Mehmet Education and Research Hospital were enrolled in this study. The patients with ophthalmic surgery and corneal pathologies other than keratoconus were excluded. All participants provided written informed consent in accordance with the Declaration of Helsinki after explanation of the nature and possible consequences of the study. Thirty patients presented with keratoconus (keratoconus group) and 30 healthy patients (control group) were included in this study. Twenty patients were using rigid gas permeable and 10 patients were not using contact lenses in the keratoconus group.

Keratoconus diagnosis was based on corneal topography and slit lamp observation. In all cases in the keratoconus group, clinical findings of keratoconus were evident: corneal topography revealing an asymmetric bowtie pattern, with or without skewed axes and at least one keratoconus sign on slit lamp examination, such as stromal thinning, conical protrusion of the cornea at the apex, Fleischer ring, Vogt striae, or anterior stromal scar.

We performed complete ophthalmological examination and corneal topography to all patients and determined their high and low contrast visual acuity and simulated *K* (sim *K*) values. Visual acuity testing was performed with Snellen and logMAR for each eye separately. Low contrast visual acuity was measured with Bailey-Lovie low contrast sensitivity chart as the maximum number of letters that patients can read. Corneal topography was performed with Nidek Magellan Mapper and corneal curvature was calculated as the average of right and left eyes steep sim *K* keratometry. The patients in the keratoconus group were graded according to the Amsler-Krumeich keratoconus classification system as grades 1 to 4.

The eyes of the patients in the keratoconus group were designated as better or worse on the basis of his or her logMAR visual acuity scores. For those with bilateral involvement, worse eyes were defined as those with a 0.1 logMAR unit or worse logMAR visual acuity score than the other eye. If both eyes of a patient had identical logMAR visual acuities, then both eyes were designated as better eyes for purposes of analyses. In patients with unilateral involvement, the visual acuity of the eye with involvement was grouped under worse eyes [14].

A validated version of NEI-VFQ-25 in Turkish was administered to each subject [4, 5]. NEI-VFQ-25 consists of 25 core and 13 optional items. It is divided into 12 subscales as general health, general vision, ocular pain, near vision, distance vision, vision-specific social functioning, vision-specific mental health, vision-specific role difficulties, vision specific dependency, driving, color vision, and peripheral vision. Questionnaire subscales were graded between 0 and 100 with higher scores representing better function. Driving question was excluded because of a large proportion of nondrivers in the study.

### 2.1. Statistical Analysis

A mean for the overall NEI-VFQ-25 score and for each subscale score was computed. The mean values of the scores within the ordered categorical variables (keratoconus category, visual acuity ≥0.4 or <0.4 in the better or worse eye, and contact lens use) were calculated. Descriptive analyses were presented as mean ± standard deviation. Scale scores were compared with Mann-Whitney *U* test and Kruskal Wallis test. The degree of linear agreement between parameters was calculated using the Spearman correlation coefficient. All statistical analyses were performed with SPSS 16.0 program. *P* value < 0.05 was considered as statistically significant.

## 3. Results

Sixty patients were included in the study. Demographic characteristics of the patients (age, gender, education, and contact lens use) are presented in Table 1. We did not observe a significant difference for age, gender, and education level between the groups (*P* > 0.05).

Best corrected binocular mean visual acuity was worse in the keratoconus group both in the better eye and worse eye when compared to the control group (*P* = 0.001). Low contrast visual acuity was also worse in the keratoconus group when compared to the control group (*P* = 0.001).

Average of the steep *K* values of both eyes was 52.9 ± 5.7 D for keratoconus group and 43.1 ± 1.9 D for the control group (*P* = 0.00). Keratoconus group was graded according to the Amsler-Krumeich keratoconus classification as stage I: 4 eyes (13.3%); stage II: 14 eyes (46.6%); stage III: 6 eyes (20.0%); stage IV: 6 eyes (20.0%). Keratoconus was unilateral in 9 patients (30%) and bilateral in 21 patients (70%). In the keratoconus group, best corrected mean visual acuity of both eyes was significantly better in the patients using contact lenses than noncontact lens users (*P* = 0.028).

All subscales of NEI-VFQ-25 were lower in the keratoconus group compared to control (Table 2). The difference was more evident in the subscales of general vision, ocular pain, near vision, vision-specific mental health, vision-specific role difficulties, and peripheral vision (*P* < 0.05). Overall composite scores were 75.2 ± 17.2 in the keratoconus group and 93.2 ± 5.6 in the control group (*P* = 0.00).

When we compared NEI-VFQ-25 subscale item scores between subgroups of the keratoconus group, NEI-VFQ-25 subscale scores were lower in the grade 4 patients compared to grade 1 keratoconus patients but the difference between the keratoconus grades was not statistically significant (*P* > 0.05). Overall composite scores were 82.6 ± 12.7, 77 ± 15.9, 72.7 ± 17.6, and 67.8 ± 25.1 for keratoconus grades I, II, III, and IV, respectively. Overall composite scores decreased with advancing keratoconus grades although the decrease was not statistically significant (*P* > 0.05).

The NEI-VFQ-25 subscale item scores showed statistically significant differences according to the visual acuity in the better eye. Patients with low visual acuity (logMAR visual acuity of 0,4 or worse) in the better eye had significantly lower distance vision, social functioning, mental health, role difficulties, and overall composite score (*P* < 0.05). Meanwhile, patients with low visual acuity (logMAR visual acuity of 0.4 or worse) in the worse eye had significantly lower general health scores compared to patients with high visual acuity.

Contact lens wearers had better best corrected visual acuity in comparison with non-contact lens wearers (*P* = 0.028). Although not significantly different (*P* > 0.05), the NEI-VFQ-25 subscale scores of distance vision, vision-specific mental health, role difficulties, social functioning, and dependency, color vision subscale scores of the patients using contact lenses were better than the patients who were not using contact lenses. Meanwhile general vision, ocular pain, near vision, and peripheral vision scores were worse in the patients using contact lenses compared to the patients who were not using contact lenses (*P* > 0.05) (Table 3).

During the correlation analysis, keratoconus grades negatively correlated with vision in the best eye and keratometry value. No significant correlation was observed between keratoconus grades and NEI-VFQ-25 subscales (*P* > 0.05). Visual acuity in the better eye correlated with near vision (*P* = 0.002; *r* = 0.547), distance vision (*P* = 0.0; *r* = 0.615), social functioning (*P* = 0.0; *r* = 0.653), vision-specific mental health (*P* = 0.0; *r* = 0.663), role difficulties (*P* = 0.001; *r* = 0.598), dependency (*P* = 0.001; *r* = 0.584), color vision (*P* = 0.007; *r* = 0.490) and peripheral vision (*P* = 0.003; *r* = 0.531), and total score (*P* = 0.0; *r* = 0.699). While, visual acuity in the worse eye correlated with general health (*P* = 0.024; *r* = 0.425), general vision (*P* = 0.01; *r* = 0.472), social function (*P* = 0.038; *r* = 0.387), mental health (*P* = 0.007; *r* = 0.490), role difficulties (*P* = 0.025; *r* = 0.416), and total score (*P* = 0.007; *r* = 0.493). No correlation was observed between the low contrast visual acuity and the parameters tested (*P* > 0.05).

Education level showed a negative correlation with social function (*P* = 0.02; *r* = 0.496), dependency (*P* = 0.02; *r* = −0.516), color vision (*P* = 0.006; *r* = −0.594), and peripheral vision (*P* = 0.02; *r* = 0.498).

## 4. Discussion

Keratoconus is an ectatic corneal disorder characterized by progressive corneal thinning that results in corneal protrusion, irregular astigmatism, and decreased vision. Despite intensive clinical and laboratory investigation, the etiology of keratoconus remains unclear. Classic histopathologic features include stromal thinning, iron deposition in the epithelial basement membrane, and breaks in Bowman's layer. Contact lenses are the most common treatment modality. When contact lenses fail, corneal transplant is the best and most successful surgical option [1].

NEI-VFQ-25 is a vision-targeted questionnaire; meanwhile it includes items that capture concerns about the future, fear, and anxiety [10, 22]. The multidimensional nature of the NEI-VFQ-25 subscales is designed to capture the impact of visual problems on physical functioning, emotional well-being, and social functioning.

VR-QoL scores may be expected to be different in the keratoconus patients from the normal people. There are a few previous reports about the impact of keratoconus on NEI-VFQ-25 scales. Collaborative longitudinal evaluation of keratoconus (CLEK) study [21] examined the VR-QoL of keratoconus patients. Their results showed that binocular entrance visual acuity worse than 20/40 was associated with lower VR-QoL scores on all scales except general health and ocular pain. A steep keratometric reading (average of both eyes) >52 D was associated with lower scores on the mental health, role difficulty, driving, dependency, and ocular pain scales. Scores for CLEK patients on all scales were between patients with category 3 and category 4 except general health, which was better than AMD patients, and ocular pain, which was worse than AMD patients. Keratoconus is associated with significantly impaired VR-QoL that continues to decline over time. CLEK patients were followed up for seven years and estimated modest decline in all scales except ocular pain and mental health. A 10-letter decline in high-contrast binocular visual acuity or a 3.00 D increase in corneal curvature was associated with significantly larger declines [23].

Tatematsu-Ogawa et al. [24] evaluated VR-QoL in 45 Japanese keratoconus patients by using the Japanese version of the NEI-VFQ-25. In patients with keratoconus including those with normal visual acuity, all NEI-VFQ-25 subscale scores were significantly lower (*P* < 0.05) than the control subjects. Subscales evaluating general health, ocular pain, and vision-specific mental health showed particularly low values. Among patients with keratoconus, every subscale score other than color vision correlated with corrected visual acuity.

In our study, all subscales of NEI-VFQ-25 were lower in the keratoconus group similar to previous studies. The difference was more evident in the subscales of general vision, ocular pain, near vision, vision-specific mental health, vision-specific role difficulties, and peripheral vision (*P* < 0.05). Overall composite score was 75.2 ± 17.2 in the keratoconus group and 93.2 ± 5.6 in the control group (*P* = 0.00).

The NEI-VFQ-25 subscale item scores did not show significant differences according to keratoconus grades, contact lens use, and gender, while the visual acuity showed significant differences. Patients with low visual acuity (logMAR visual acuity of 0.4 or worse) in the better eye had significantly lower distance vision, social functioning, mental health, role difficulties, and overall composite score (*P* < 0.05). Meanwhile, patients with low visual acuity (logMAR visual acuity of 0.4 or worse) in the worse eye had significantly lower general health scores compared to patients with high visual acuity (logMAR visual acuity of 0.3 or better).

In our study, when the sample was divided into mild (grade I), moderate (grade II), and severe (grades III and IV) keratoconus cases, overall composite scores were 82.6 ± 12.7, 77.0 ± 15.9, 72.7 ± 17.6, and 67.8 ± 25.1 for keratoconus grades I, II, III, and IV, respectively. Overall composite scores seem to be decreased with advancing keratoconus grades, although our study contain only a small number of patients in each in each keratoconus group to obtain significative conclusions.

The general health question is treated as a stand-alone-list-item, because it is a robust marker of overall health status in many population-based studies and provides a comparative benchmark for groups of persons who complete the NEI-VFQ-25. In our study, general health score was 65.0 ± 20.6 in keratoconus group and 79.7 ± 15.8 in control group (*P* = 0.030). Interestingly, vision in the worse eye ≥0.4 logMAR had a significantly decreased general health score compared to patients with <0.4 logMAR vision.

NEI-VFQ results revealed differences in gender in self-reported difficulty with distance activities and driving in CLEK study. Women were more likely than men to report ocular symptoms of dryness and complaints based upon a composite score of ocular symptoms. Gender differences may exist in patient history, vision, and ocular symptoms in keratoconus patients [25].

In keratoconus patients who have undergone penetrating keratoplasty in one or both eyes VR-QoL remains impaired despite satisfactory results on visual outcome measures obtained [26]. Significantly lower NEI-VFQ scores are reported in postkeratoplasty keratoconus patients compared to CLEK historical control group for the subscales of role difficulties, dependency, driving, and peripheral vision. In general, scores of that study were between scores of patients with AMD categories 3 and 4. Patients with visual acuity better than 20/40 (in the better eye) showed significantly higher scores in all subscales except color vision [15].

It has been shown that keratoconus exerts a significant impact on keratoconus patients' VR-QoL, even in its early stages (grade I) with normal best-spectacle-corrected visual acuity compared to contact lens users without keratoconus. Corneal collagen crosslinking (CXL) and CXL combined with topography-guided photorefractive keratectomy (tCXL) have been shown to exert a beneficial impact on self-reported VR-QoL. Significant differences were detected in “mental health” and “dependency” VFQ-25 domains for both the CXL and tCXL groups (*P* = 0.05). Furthermore, the tCXL group demonstrated significant differences in the “near activities” (*P* = 0.04), “role limitations” (*P* = 0.02), and “driving” (*P* < 0.01) subscale scores in a study by Labiris et al. [27].

Contact lenses are one of the better solutions to correct refractive errors induced by keratoconus. Contact lens fitting on a conical cornea smooths out the highly irregular optical surface of the cornea and improves visual acuity considerably. The quality and quantity of vision is far better than with spectacle lens correction [28]. Success in the contact lens usage in the keratoconus patients may increase the visual acuity and vision related quality of life. In a study evaluating keratoconus patients using rigid gas permeable lenses, the NEI-VFQ-25 overall score was 79.2 and keratoconus was associated with lower scores in dependency, mental health, and ocular pain categories [29]. In our study, NEI-VFQ-25 overall score was 75.4 in the keratoconus group of contact lens users. Although not significantly different (*P* > 0.05), the NEI-VFQ-25 subscale scores of distance vision, vision-specific mental health, role difficulties, social functioning and dependency, and color vision subscale scores of the patients using contact lenses were better than the patients who do not use contact lenses. Meanwhile general vision, ocular pain, near vision, and peripheral vision scores were worse in the patients using contact lenses (*P* > 0.05).

Lee et al. [30] has demonstrated previously the importance of eye symptoms to general HR-QoL using the short form-36 version. Trouble seeing and blurred vision both had statistically unique associations with worse scores on the HR-QoL, while the presence of eye diseases, such as glaucoma, cataract, and macular degeneration, did not have an association after adjusting for other variables in the model. Similarly, vision in the better eye was the most significant parameter affecting VR-QoL in the keratoconus group of patients in our study.

Main limitation of our study is small sample size. Large sized prospective studies comparing treatment options of keratoconus including different types of contact lenses may be helpful in the future. As a conclusion, keratoconus patients have a decreased vision related quality of life as demonstrated by the NEI-VFQ-25 when compared to the controls. As the patients with keratoconus are generally young adults in their active years, to understand their concerns about their future is an important public health aspect that can be used to modify their treatments.

Our results suggest that vision in the best eye is the most important parameter affecting VR-QoL of the patients with keratoconus. Keratoconus patients should maintain their best vision that they can have. Success in the contact lens usage and maintaining higher visual acuity in the keratoconus patients may improve VR-QoL.

## Figures and Tables

**Table 1:** Demographic characteristics of the patients (age, gender, education, and contact lens use and high contrast (Snellen) and low contrast visual acuity (letters)) according to the groups (*P < 0.05; **P < 0.001).

	Keratoconus group	Control group	*P*
Age	29.36 ± 10.60	30.23 ± 8.80	0.573
Gender			
Female/male	16/14 (53/47%)	18/12 (600/40%)	0.916
Education level			
Primary school	3 (10%)	6 (20%)	0.562
High school	20 (67%)	12 (40%)	
University	7 (23%)	12 (40%)	
Contact lens wear			
None	10 (33%)	30	0.00**
One eye only	5 (17%)		
Both eyes	15 (50%)		
Visual acuity			
Better eye	0.73 ± 0.23 (0.21 ± 0.23 LogMAR)	1.0 (0.0 logMAR)	0.00**
Worse eye	0.47 ± 0.27 (0.4 ± 0.33 LogMAR)	1.0 (0.0 logMAR)	0.001**
Low contrast visual acuity	23.27 ± 11.28 (0.54 ± 0.22 LogMAR)	45.00 ± 3.20 (0.10 ± 0.08 LogMAR)	0.001**

**Table 2:** NEI-VFQ-25 subscale scores according to the groups (*P < 0.05; **P < 0.001).

NEI-VFQ-25 scales	Keratoconus group	Control group	*P*
General health	65.0 ± 20.6	79.7 ± 15.8	0.030*
General vision	60.2 ± 24.4	89.7 ± 10.3	0.001**
Ocular pain	54.0 ± 23.8	78.8 ± 17.9	0.002*
Near vision	76.0 ± 23.0	93.5 ± 13.6	0.014**
Distance vision	84.0 ± 18.0	94.7 ± 8.7	0.80
Social functioning	85.0 ± 24.0	98.8 ± 4.8	0.069
Mental health	67.0 ± 27.8	98.8 ± 4.8	0.00**
Role difficulties	77.2 ± 26.4	96.4 ± 7.8	0.014*
Dependency	84.7 ± 26.4	96.4 ± 7.8	0.379
Color vision	91.0 ± 17.0	97.6 ± 6.6	0.424
Peripheral vision	80.7 ± 17.4	94.1 ± 9.3	0.020*
Overall composite score	75.2 ± 17.2	93.2 ± 5.6	0.00**

**Table 3:** Comparison of NEI-VFQ-25 subscale item scores in the keratoconus group according to the subgroups of the keratoconus grade; visual acuity in the better and worse eye and contact lens use are presented (*P < 0.05; **P < 0.001).

NEI-VFQ-25 scores	Keratoconus grade					Visual acuity, better eye			Visual acuity, worse eye			Contact lens use		
	I *n*: 6	II *n*: 9	III *n*: 7	IV *n*: 6	*P*	≥0.4 *n*: 5	<0.4 *n*: 25	*P*	≥0.4 *n*: 13	<0.4 *n*: 17	*P*	Yes *n*: 20	No *n*: 10	*P*
General health ±SD	66.6 15.2	67.7 21.0	53.7 26.8	70 20	0.771	52.5 12.5	69.6 21.5	0.102	54.4 12.3	77.2 21.9	0.013*	65 27.8	65 18.7	0.849
General vision ±SD	60.0 17.3	70 21.6	35 12.2	65 35	0.112	68.7 33.7	59.2 22.5	0.487	60.5 26.1	62.2 24.5	0.859	49 20.1	64.2 25.1	0.284
Ocular pain ±SD	60.0 20	51.1 27.01	48.7 23.2	61.2 25.9	0.774	59 23	53.2 25.3	0.604	59 25.4	50 23.3	0.454	46 31.1	56.6 21.5	0.351
Near vision ±SD	83.3 28.8	82.2 16.2	62.5 25	71.2 32.2	0.503	68 27.9	81 20.7	0.340	79 24.8	76.1 21.7	0.644	70 18.7	78.3 24.5	0.346
Distance vision ±SD	93.3 11.5	83.3 19.2	82.5 20.6	80 21.6	0.772	71 15.9	90.3 15.7	0.024*	83 18.1	87.7 17.8	0.517	87 13.9	83 19.6	0.891
Social functioning ±SD	100 0	81.6 26.6	93.7 12.5	72.5 32	0.371	64 25	91.4 20.42	0.030*	80 24.03	88.8 25.3	0.203	93 10.9	82.3 26.7	0.730
Mental health ±SD	66.6 11.5	72.7 32.8	65 30	56.2 28.09	0.730	38 24.6	79.2 20.1	0.008*	65 34.9	72.2 18.5	0.834	76 21.9	64 29.6	0.475
Role difficulties ±SD	83.3 15.2	75.5 27.5	81.2 37.5	72.5 27.5	0.818	53 24.3	84.2 22.8	0.022*	71 26.1	81.6 27.6	0.240	78 31.3	77 25.8	0.928
Dependency ±SD	93.3 11.5	83.8 27.8	87.5 25	75 43.3	0.975	63.7 33	89.6 23.2	0.057	83.8 27.8	83.8 27.8	1.0	90 22.3	82.8 28.3	0.536
Color vision ±SD	100 0	88.3 20.6	93.7 12.5	87.5 25	0.750	86 21.9	92.1 17.5	0.51	90.5 17	90.5 20.6	0.83	95 11.1	89.6 19.8	0.731
Peripheral vision ±SD	86.6 23	83.8 16.9	77.5 20.6	72.5 25	0.660	74 19.4	83.2 17.4	0.358	82.5 17.8	78.8 19	0.73	74 13.4	83 18.4	0.315
Overall composite score ±SD	82.6 12.7	77 15.9	72.7 17.6	67.8 25.1	0.682	61.9 17.7	80.2 15.4	0.042*	74 17.6	77 18.5	0.462	75.4 15.9	75.1 18.1	0.760
